# Therapeutic Drug Monitoring and Pharmacogenetic Testing as Guides to Psychotropic Drug Dose Adjustment: An Observational Study

**DOI:** 10.3390/ph17010021

**Published:** 2023-12-22

**Authors:** Elodie Cuvelier, Houda Khazri, Cloé Lecluse, Benjamin Hennart, Ali Amad, Jean Roche, Michel Tod, Guillaume Vaiva, Olivier Cottencin, Pascal Odou, Delphine Allorge, Bertrand Décaudin, Nicolas Simon

**Affiliations:** 1 CHU Lille, Institut de Pharmacie, F-59000 Lille, Francepascal.odou@univ-lille.fr (P.O.); bertrand.decaudin@univ-lille.fr (B.D.); nicolas.simon@univ-lille.fr (N.S.); 2 GRITA—Groupe de Recherche Sur Les Formes Injectables Et Les Technologies Associées ULR 7365, CHU Lille, University Lille, F-59000 Lille, France; 3 CHU Lille, Pôle de Biologie-Pathologie-Génétique, Unité Fonctionnelle de Toxicologie, F-59000 Lille, France; benjamin.hennart@chu-lille.fr (B.H.); delphine.allorge@univ-lille.fr (D.A.); 4 Inserm, CHU Lille, U1172—LilNcog—Lille Neuroscience & Cognition, University Lille, F-59000 Lille, France; ali.amad@chu-lille.fr (A.A.); guillaume.vaiva@chu-lille.fr (G.V.); 5 CHU de Lille, Unité de Psychogériatrie, Pôle de Gérontologie, F-59037 Lille, France; jean.roche@chu-lille.fr; 6 UMR 5558, Laboratoire de Biométrie et Biologie Évolutive, Université Lyon 1, F-69622 Lyon, France; michel.tod@univ-lyon1.fr; 7 CHU de Lille, Service d’addictologie, CNRS, UMR 9193, SCALab, équipe psyCHIC, CS 70001, Université de Lille, F-59037 Lille, France; olivier.cottencin@chu-lille.fr; 8 CHU Lille, Institut Pasteur Lille, ULR 4483—IMPECS—IMPact de l’Environnement Chimique sur la Santé Humaine, Université de Lille, F-59000 Lille, France

**Keywords:** pharmacogenetics, psychiatry, clinical decision-making tool, therapeutic drug monitoring

## Abstract

To avoid the failures in therapy with psychotropic drugs, treatments can be personalized by applying the results of therapeutic drug monitoring and pharmacogenetic testing. The objective of the present single-center observational study was to describe the changes in psychotropic drug management prompted by therapeutic drug monitoring and pharmacogenetic testing, and to compare the effective drug concentration based on metabolic status with the dose predicted using an in silico decision tool for drug–drug interactions. The study was conducted in psychiatry wards at Lille University Hospital (Lille, France) between 2016 and 2020. Patients with data for at least one therapeutic drug monitoring session or pharmacogenetic test were included. Blood tests were performed for 490 inpatients (mainly indicated by treatment monitoring or failure) and mainly concerned clozapine (21.4%) and quetiapine (13.7%). Of the 617 initial therapeutic drug monitoring tests, 245 (40%) complied with good sampling practice. Of the patients, 51% had a drug concentration within the therapeutic range. Regardless of the drug concentration, the drug management did not change in 83% of cases. Thirty patients underwent pharmacogenetic testing (twenty-seven had also undergone therapeutic drug monitoring) for treatment failure; the plasma drug concentration was outside the reference range in 93% of cases. The patient’s metabolic status explained the treatment failure in 12 cases (40%), and prompted a switch to a drug metabolized by another CYP450 pathway in 5 cases (42%). Of the six tests that could be analyzed with the in silico decision tool, all of the drug concentrations after adjustment were included in the range estimated by the tool. Knowledge of a patient’s drug concentration and metabolic status (for CYD2D6 and CYP2C19) can help clinicians to optimize psychotropic drug adjustment. Drug management can be optimized with good sampling practice, support from a multidisciplinary team (a physician, a geneticist, and clinical pharmacist), and decision support tools.

## 1. Introduction

Interindividual differences in the pharmacodynamic and pharmacokinetic factors involved in drug responses should be taken into account, in order to personalize treatment regimens [1]. These factors include poor adherence with long-term treatments and variability in cytochrome P450 (CYP) superfamily activity [2,3]. The main sources of variability in CYP activity are drug–drug interactions (DDIs), liver disorders, and polymorphisms in CYP2D6 and CYP2C19 (both of which are involved in the metabolism of many psychotropic drugs) [4,5,6,7,8].

The use of genotyping, therapeutic drug monitoring (TDM), clinical assessments, and literature reviews can improve psychotropic drug prescription and management [9]. Firstly, TDM can be used to highlight a lack of treatment adherence or the consequences of certain DDIs by detecting under- or over-exposure to a drug. This tool allows physicians to personalize drug therapies by targeting predefined plasma concentration ranges [10]. Secondly, pharmacogenetic (PG) testing can identify the patient’s metabolic capacity by revealing the presence of genetic variants, and thus define the metabolic phenotypes: extensive metabolizers (EMs, i.e., the usual phenotype), poor metabolizers (PM, with a total loss of activity), intermediate metabolizers (IMs, with a partial loss of activity), and ultrarapid metabolizers (UMs, abnormally high activity) [11]. Accordingly, the concentrations of psychotropic drugs may vary with the phenotype: the PM and IM phenotype tend to be associated with elevated drug concentrations, and the UM phenotype tends to be associated with low drug concentrations [12]. Indeed, PG testing has been described in the literature as a reliable tool for personalizing drug prescriptions in psychiatry. The application of PG testing appears to decrease the level of depressive symptoms and optimize the prescription of psychotropic (AP) drugs [9,13].

A number of guidelines on TDM and PG testing have been issued by learned societies and medical associations. The Arbeitsgemeinschaft für Neuropsychopharmakologie und Pharmakopsychiatrie (AGNP) interdisciplinary working group considers TDM to be very useful for a number of common indications: potentially poor adherence to treatment, a lack of response at therapeutic doses, suboptimal drug tolerance, patients at a high risk of drug response variability (very young and very old patients, patients with liver or kidney failure, etc.), and exposure to potential DDIs involving neuropsychiatric drugs [10,12,14]. Along with TDM, PG testing can also be used to consider interindividual variability in drug response, and thus adjust drug prescriptions to the patient’s genetic profile [15]. However, the conditions for performing TDM and PG testing depend on the guidelines followed and the clinical context—especially when the variability in the response to drug treatment may be due to a genetic factor. Although the AGNP recommends combining PG testing with TDM under specific conditions only (i.e., a drug concentration outside the standard range, despite an appropriate dose level and in the absence of obvious poor adherence or DDIs), the Clinical Pharmacogenetics Implementation Consortium and the French Réseau National de Pharmacogénétique network consider that PG testing should be conducted before a selective serotonin reuptake inhibitor is prescribed [16,17,18].

There are also guidelines and tools to help physicians interpret PG data and integrate the latter into their clinical practice. Firstly, the AGNP guidelines and the Dutch Pharmacogenetics Working Group have developed a tool for psychotropic drug dose adjustment as a function of the patient’s metabolic status (as determined by PG testing) [15,16]. Secondly, DDI-Predictor (www.ddi-predictor.org, accessed on 22 January 2021) is a free online decision-making tool that characterizes pharmacokinetic modulations involving the main CYPs, and helps to adjust the dose level as a function of the patient’s metabolic status for CYP450 [16,17,18]. It has been reported that DDI-Predictor can help pharmacists to resolve medication issues in the event of a DDI [19].

In view of these various guidelines and tools, we have sought to optimize psychotropic drug prescribing in our hospital. In particular, we want to understand the value of TDM and PG testing for guiding our care strategy. Hence, the primary objective of the present study was to describe the management of psychiatric patients with treatment failure, from hospital admission to clinical improvement. The secondary objective was to analyze DDI-Predictor’s ability to determine the effective dose of a psychotropic drug prescribed to a patient before PG testing.

## 2. Results

### 2.1. Characteristics of the Study Population

**Figure 1:**
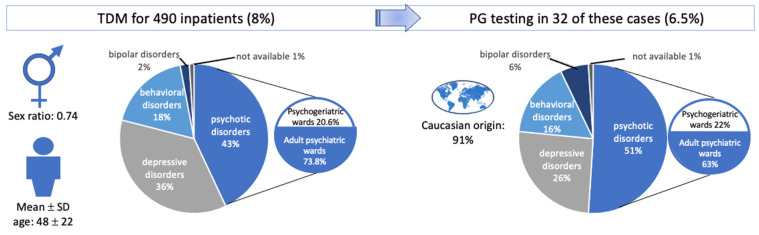
Description of the study population having undergone TDM and PG testing.

Between 2016 and 2020, 490 of the 5816 inpatients (8%) underwent a total of 1287 TDM sessions (Figure 1). The mean length of hospital stay was 70 days (range: 1–8412 days; median: 25 days). Twenty-four patients underwent TDM in a day hospital setting because our hospital is a psychiatric referral centre. The mean time interval between admission and the first TDM was 31 days (range: 1–6795 days; median: 3 days). The turnaround times for the TDM and PG results were 2 to 3 days and 15 days to 17 months, respectively. According to the guidelines issued by psychiatry associations, TDM should be performed no sooner than two weeks after a dose level change. Therapeutic optimization lasted for 50 to 65 days after TDM testing, and for 130 to 150 days after PG testing (i.e., when the correct dose level is obtained after one or two adjustments). Patients were discharged from hospital when they appeared to be clinically stable or improved.

### 2.2. The Drug Management Process after TDM

During a given hospital stay, a patient underwent TDM between 1 and 20 times. Each TDM session primarily involved one drug, although some involved two or three drugs. Most of the blood samples were taken at steady state (57%) and at the recommended time (59%), i.e., at the trough or just before the next dose. A total of seven APs were measured: clozapine and *N*-desmethylclozapine (*n* = 276; 21.4%), quetiapine (*n* = 176; 13.7%), risperidone and 9-hydroxy-risperidone (*n* = 134; 10.4%), olanzapine (*n* = 111; 8.6%), aripiprazole and dehydro-aripiprazole (*n* = 88; 6.9%), amisulpride (*n* = 71; 5.5%), and haloperidol (*n* = 20; 1.6%). Nine ATDs were assayed: sertraline (*n* = 119; 9.2%), paroxetine (*n* = 69; 5.4%), clomipramine and *N*-desmethylclomipramine (*n* = 57; 4.4%), mirtazapine (*n* = 48; 3.7%), venlafaxine and *O*-desmethylvenlafaxine (*n* = 45; 3.5%), escitalopram and *N*-desmethylescitalopram (*n* = 43; 3.3%), fluoxetine and norfluoxetine (*n* = 14; 1.1%), amitriptyline and nortriptyline (*n* = 10; 0.8%), and duloxetine (*n* = 6; 0.5%). With the except of aripiprazole, both the active drug and the above-mentioned metabolite were assayed. Aripiprazole alone was assayed in 45 of the 88 TDM sessions (51%).

The justifications for TDM were a risk of a variable response (APs: 58%; ATDs: 66%), a lack of response at therapeutic doses (APs: 31%; ATDs: 29%), intolerance (APs: 7%; ATDs: 3%), or uncertain adherence to treatment (APs: 8%; ATDs: 2%).

**Figure 2:**
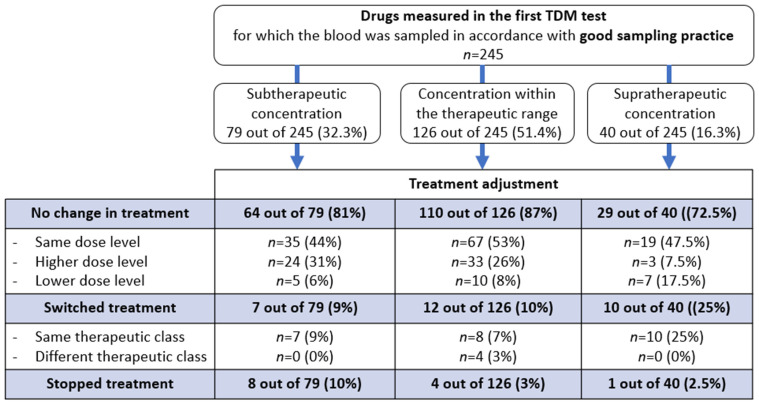
Drug management as a function of the plasma concentration of the initially prescribed psychotropic drug in the first therapeutic drug monitoring (TDM) session.

Among the first documented TDM results (617 out of 761), 245 (40%) were performed in line with good practice (i.e., at the recommended time and at the steady state). The TDM result did not prompt a change in the initially prescribed drug in 203 of the 245 cases (83%). The drug was switched in 11.8% of cases and withdrawn in 5.3% (Figure 2). 

### 2.3. The Drug Management Process after PG Testing

Thirty-two patients were genotyped for the genes encoding CYP2D6 (*n* = 26), CYP3A5 (*n* = 10), CYP3A4 (*n* = 10), CYP1A2 (*n* = 10), CYP2C19 (*n* = 5), CYP2C9 (*n* = 1), and/or CYP2B6 (*n* = 1). Two of the thirty-two patients were ultimately excluded from our analysis because their psychotropic drugs (paliperidone and citalopram) were administered intramuscularly and not per os. Twenty-seven of the thirty patients (90%) were only genotyped once.

Among the nine psychotropic drugs taken by the genotyped patients, there were five Aps (*n* = 7 for quetiapine, *n* = 6 for clozapine, *n* = 6 for risperidone, *n* = 2 for aripiprazole, and *n* = 2 for olanzapine) and four ATDs (*n* = 5 for paroxetine, *n* = 3 for fluoxetine, *n* = 1 for escitalopram, and *n* = 1 for venlafaxine). The major CYP450 isoforms involved in the metabolism of these drugs are CYP2D6 (for 3 of the 9 drugs), CYP2C19 (2 drugs), CYP1A2 (2 drugs), and CYP3A4 (2 drugs). Isoforms of CYP450 involved in the metabolism of psychotropic drugs are described in Supplementary Data.

Twenty-five of the thirty genotyped patients (90%) had undergone TDM for various reasons: a lack of a treatment response at therapeutic doses (*n* = 18; 67%), potentially poor adherence (*n* = 5; 18%), or suboptimal tolerance (*n* = 4; 15%) (Table 1). Among the 5 (18%) patients with potentially poor adherence, TDM was repeated in 4 cases and confirmed this hypothesis. In 23 of the 25 cases (93%), the drug concentration was outside the reference range.

No other confounding pharmacokinetic factors involved in variability of drug response (kidney failure, liver failure, and DDIs) were observed. One of the eight patients treated with clozapine or olanzapine had a likely interaction with tobacco (consumption > 10 cigarettes/day).

In order to understand the persistence of treatment failure after dose modification following the TDM, PG testing was performed.

The drug adjustments according to the metabolic status are described in Table 2. The EM phenotype corresponds to a normal phenotype for all CYP450 isoforms (including genotype *1/*1F of CYP1A2 in non-smoker patients) other than CYP3A5; for the latter enzyme, the majority (90%) of Caucasians lack full activity and are considered to be PMs. In 17 (57%) of the 30 cases, the drug or drug level was adjusted before the PG test results had been delivered. The presence of one or more genetic variations (in 18 of the 30 patients (60%)) may have been responsible for treatment failure in 12 cases (40%). Changes in medication were observed for 10 of these 12 patients (83%): 5 drug switches (50%), 4 dose adjustments (40%), and 1 dosing schedule adjustment (10%).

### 2.4. Use of the DDI-Predictor Tool to Adjust the Psychotropic Drug Regimen to the Patient’s Metabolic Status

Eleven of the twenty-six patient genotypes for CYP2D6 continued to receive the same drug. The CYP2D6 genotype was identified by DDI-Predictor in nine cases. In three of these cases, the drug concentration could have been modulated by other polymorphisms, which could have led to interpretation bias. Ultimately, only 6 of the 30 patients (20%) were selected for further analysis (Table 3). The effective drug concentration after adjustment was included within R_AUC_ range estimated by DDI-Predictor in all six patients. Hence, dose level adjustments based on a clinical assessment (expressed by the R_d_ ratio) were consistent with the doses predicted by DDI-Predictor (i.e., the R_AUC_).

## 3. Discussion

In order to optimize drug management for patients in adult psychiatry or geriatric psychiatry wards, the main objective of this study was to describe the reasons for the use of TDM and PG testing in a university hospital where these tests are routinely used.

The patients showed treatment failure and/or risk factors for a variable drug response. Follow-up appears to be necessary because the TDM result was outside the reference therapeutic range in just under half of the cases. However, our study results show that 68% of the TDM results could not be interpreted because of a lack of information on the sampling technique (144 out of 761; 19%) or a lack of compliance with good sampling practice (372 out of 761; 49%). Given that misinterpretation of these tests leads to erroneous drug adjustments in 20% of cases [20,21], it is necessary to optimize this step by training healthcare professionals in good sampling practice.

Interestingly, the TDM result did not usually change the initially prescribed psychotropic drug. An initial subtherapeutic plasma concentration could be ascribed to poor adherence: four patients in this situation had a second TDM session a few days later, after drug intake had been checked carefully by the hospital staff. For the other 31 patients with a subtherapeutic plasma concentration, this lack of a drug change after TDM may be due to intrinsic patient factors (e.g., age, weight, and poor drug absorption) and extrinsic factors (such as DDIs). In a patient with a supratherapeutic concentration, the maintenance of the initial treatment can be justified when (i) the concentration is below the toxic threshold and (ii) the benefit/risk balance is favorable for the patient (i.e., a good tolerance and an improvement in clinical status). Our results suggest that the interpretation of TDM results by a medical biologist requires multidisciplinary collaboration with a physician who can relate this interpretation to the patient’s clinical status and medication history [10]. When analyzing prescriptions and participating in multidisciplinary staff meetings, a clinical pharmacist can help to determine the cause of treatment failure and improve the application of TDM [22]. By analyzing the dose-related concentration and the metabolite-to-parent compound ratio defined in the 2017 AGNP guideline, both the medical biologist and clinical pharmacist can help to optimize the interpretation of TDM results by considering poor adherence, a DDI, or a genetic abnormality in drug metabolism [10].

Another problem relates to inter-study differences in the guidelines followed, the indications for PG testing, and interpretation of the test results [23]. Our hospital is a regional reference center for PG testing, in line with the AGNP guidelines [15]. However, the organization of PG testing could still be improved because the turnaround time can sometimes be long and may affect drug optimization. However, the latest generation of genotyping platforms for various CYPs has a turnaround time of 2 to 3 weeks. Multidisciplinary meetings should be held as soon as the TDM results are obtained. The medical biologist’s expert knowledge can help in the choice of the CYPs to be genotyped; then, knowledge of the patient’s metabolic status can enable the prescribing physician to optimize drug treatment rapidly. However, systematic genotyping (regardless of the patient’s drug history) may shorten the turnaround time for the results, and thus also enable optimization of the treatment choice. For compliant patients, testing could perhaps be performed 4 weeks after treatment initiation, with (i) TDM in the event of a moderate treatment response, and with (ii) TDM and PG testing in the event of treatment failure.

A PG test was requested when the TDM revealed an unexpected drug concentration; according to the physician, there was no obvious explanation for the clinical effect in 30 of the 459 cases. Interestingly, most of the requests for PG tests concerned CYP2D6 or CYP2C19, both of which metabolize the majority of psychotropic drugs and are influenced by genetic polymorphisms [5]. Obtaining the results was essential because (i) at least one genetic mutation was detected in 60% of cases; (ii) variability in drug response could be explained by the patient’s metabolic status in 58% of cases; and (ii) another medication was prescribed in 68% of cases. Given the occasional long turnaround times, the drug regimen is sometimes modified before the PG results are obtained; in an emergency, it may be appropriate to prescribe a psychotropic drug metabolized by another metabolic pathway. However, the CYP2D6 phenotype was analyzed most often (93%), and the gene encoding CYP2C19 (known for its multiple functional variants) was analyzed in only 7% of cases. Given that a drug metabolized by CYP2D6 was switched for a drug metabolized by CYP2C19 in 28% of the cases in our study, systematic screening for *CYP2D6* and *CYP2C19* variants may enable drug prescriptions to be optimized (as suggested in the literature [13]. Our hospital’s current new genotyping platform can test for various CYP isoforms (such as 2D6, 2C19 and 3A4/5) in a single run.

The preliminary results for DDI-Predictor (a tool that can assist the physician to prescribe psychotropic drugs as a function of the patient’s metabolic status) were positive because the dose level adjustments based on a clinical assessment were consistent with the predicted plasma concentrations in all cases. DDI-Predictor could be used to systematically calculate the potentially effective dose as soon as the PG test results becomes available (e.g., 2 weeks later). Nevertheless, DDI-Predictor can only be applied under certain conditions, which explains the low number of patients included in this part of the study (6 out of 30; 20%). The analysis targeted certain CYP genotypes and did not take drug response variability factors other than DDIs. The tool would have to be improved for more precise dose adjustment in routine clinical practice.

The study had a number of limitations. Firstly, the lack of data on long-term therapeutic adjustment limited the interpretation of patient management approaches. Secondly, non-compliant blood sampling limited the interpretation of half of the TDM tests. Thirdly, the time interval between each step for dose adjustment and the involvement of the various staff (e.g., medical biologists and pharmacists) was sometimes difficult to determine on the basis of hospital discharge letters. Lastly, our retrospective analysis of TDM and PG testing prevented us from comparing treatment changes in tested vs. non-tested patients. It would be interesting to address the questions of personalized treatment and the systematic implementation of a multidisciplinary team meeting in a prospective study.

## 4. Materials and Methods

### 4.1. Study Design

In a retrospective, observational, single-center study, we assessed patients admitted to any of the 10 psychiatry wards (corresponding to a total of 123 beds) at Lille University Hospital (Lille, France) between 1 January 2016, and 31 December 2020, and who had available data related to TDM.

### 4.2. Study Context

**Figure 3:**
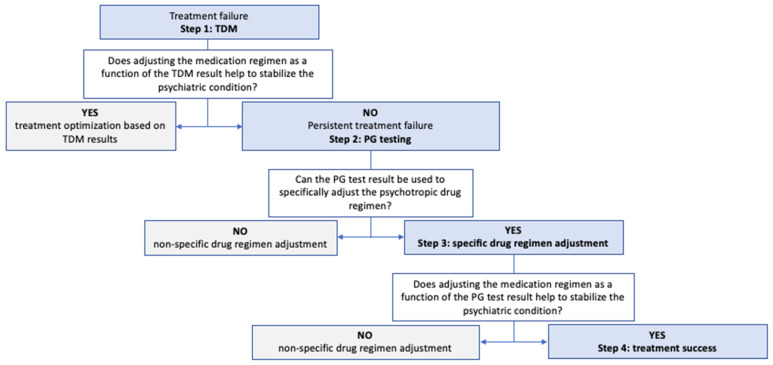
Management of patients hospitalized for apparent treatment failure. The factors involved in drug response variability are sought in a stepwise manner, with a view to improve the effectiveness of treatment. TDM: therapeutic drug monitoring; PG: pharmacogenetic.

Once a psychiatric disease has been diagnosed, drug therapy can be initiated or reassessed. In some cases, patients with treatment failure or poor tolerance are admitted to hospital, in order optimize their drug regimen (Figure 3). Based on the patient’s clinical and laboratory work-up and medication history, TDM is performed to detect factors that may explain treatment failure: poor adherence, a DDI, or kidney or liver failure (step 1). If treatment failure is still observed one week later despite a well-adjusted treatment regimen according to the TDM results (good adherence, and a supposedly appropriate dosing regimen), the patient’s metabolic status is assessed (with his/her consent) via PG testing (step 2). Depending on the PG results, and with advice from the medical geneticist and the clinical pharmacist, the physician can then adjust the drug regimen (a dose level adjustment, a dosing frequency adjustment, or a drug switch) (step 3). Lastly, the treatment is considered to be effective if the last change in the drug dosing regimen is associated with an improvement in the patient’s signs and symptoms (step 4). Our hospital’s central laboratory routinely provides PG testing for several CYP450, including the isoforms CYP1A2, 2B6, 2D6, 2C9, 2C19, 3A4, and 3A5 involved in the metabolism of the main psychotropic drugs authorized in France (Supplementary Data).

### 4.3. Therapeutic Drug Monitoring and PG Testing

Therapeutic drug monitoring measured the patients’ residual plasma levels in the morning, before drug administration treatment. Briefly, antipsychotics and antidepressants (ATDs) were assayed in 50 µL samples of serum extracted using 200 µL of acetonitrile containing 1 mg/L deuterated internal standards (i.e., olanzapine-D8, risperidone-D4, haloperidol-D4, and clozapine-D4 for APs; sertraline-D3, duloxetine-D3, and venlafaxine-D4 for ATDs). The samples were centrifuged (10 min, 17,000× *g*, 4 °C), and the supernatants (20 µL) were added to 180 µL of deionized water containing 0.1% formic acid and 5 mM ammonium formate. A volume of 5 µL of the mixture was injected onto an ultraperformance liquid chromatography/tandem mass spectrometry system (Acquity TQ-D Detector, Waters, Milford, MA, USA) equipped with a HSS C18 column. Ions of each analyzed compound were detected in positive ion mode, using multiple reaction monitoring.

For the PG testing, DNA was extracted from 1 mL of total blood using a Perkin-Elmer/B2K extraction kit and a Chemagic-Star robot DNA extractor (Hamilton Company, Reno, NV, USA, and Perkin-Elmer, Waltham, MA, USA). The quality and quantity of extracted DNA were determined using the Thermo Scientific^TM^ NanoDrop One/OneC UV-visible microvolume spectrophotometer. Primers were designed using the Fluidigm D3™ assay design web-based tool, and included all exonic regions and at least 30 base pairs of each flanking intron for a panel of genes involved in drug metabolism (CYP1A2, CYP2D6, CYP2C9, CYP2C19, CYP3A4, CYP3A5, CYP2B6, and UGT1A1 for APs and ATDs). Genomic DNA was amplified in up to 10-plex PCR reaction wells, followed by the addition of barcode indexes and sequencing adaptors via further PCR, according to the manufacturer’s instructions. The pooled amplicons were harvested and diluted to prepare unidirectional libraries for 150 base-pair paired-end sequencing on an Illumina MiSeq sequencing platform (Illumina, San Diego, CA, USA). Variants were called with MiSeq Reporter v2.6, GATK v3.7 or GATK v4.1.4.0 (Genome Analysis Toolkit). All of the very rare variants (maximum allele frequency ≤ 0.1%) and novel variants identified by next-generation sequencing were confirmed by Sanger sequencing.

### 4.4. Data Collection

Our analysis included all patients with at least one TDM result. TDM requested by the department but not carried out (because the request was canceled or the blood sample was not collected) was not considered. Furthermore, lithium assays were excluded because dose optimization depends on mandatory assays, and PG testing is of little interest (i.e., lithium is not metabolized in the liver).

The patients were selected from two inhouse databases curated by the central laboratory. The data were recorded continuously by medical biologists. The first database contained the TDM results for patients admitted to psychiatric wards between 2016 and 2020. The reference therapeutic range used by medical biologists for each result was specified in the 2011 or 2017 AGNP guidelines, depending on the date of the test [10,24]. The second database contained PG test results requested by the hospital’s psychiatry departments. All other data required for the present study (see below) were extracted from the patients’ electronic medical records.

### 4.5. Primary Objective

The following data were collected from patients’ electronic medical records; intrinsic and extrinsic factors in drug response variability were identified: physiological factors (sex and age at the time of diagnosed treatment failure), co-morbidities (liver and renal failure, undernutrition), and the presence of DDIs or other pharmacokinetic impairments (concomitant medications, or tobacco, cannabis, and/or alcohol consumption). The following descriptive data were recorded: the hospital department requesting the TDM and/or PG testing, the clinical justification for TDM and/or PG testing, and the psychotropic drug(s) initially prescribed. Specific information required for interpretation of the TDM results was sought in conjunction with interpretation of the TDM results and, if available, the PG test results: the date of drug initiation, the dose regimen, the date and time of the last administration, the date and time of blood sampling, and the presence of any prodrugs or active metabolites (good sampling practice includes both residual and steady-state sampling). With regard to therapeutic optimization, the treatment adjustment (i.e., dose adjustment, switching, or withdrawal) and the effective dose level of the psychotropic drug (as mentioned in the patient’s discharge letter) were sought. Finally, the time intervals between the four steps described in Figure 1 were estimated from the dates of the laboratory reports and hospital discharge letters.

### 4.6. Secondary Objective

The DDI-Predictor open-access decision-support tool (www.ddi-predictor.org, launched in 2013) was developed by the Genophar working group at Lyon University Hospital (Lyon, France). This software tool characterizes the pharmacokinetic changes involving the main CYPs. One of DDI-Predictor’s modules forecasts variations in drug exposure levels in patients with certain polymorphisms in the genes encoding for CYP2D6, CYP2C9, and CYP2C19, relative to the reference genotype (*1/*1). DDI-Predictor’s algorithm is based on the steady-state equations in a physiologically based pharmacokinetic model [25]. The area under the curve (AUC) data specifying the effect of CYP450 polymorphisms were obtained in population studies. In order to use the “polymorphism” module on the DDI-Predictor website, the operator has to indicate the patient’s age (< or ≥2 years), the international common name of the orally administered drug, and the patient’s metabolic status (one of the genotypes included on the module) [18,21]. DDI-Predictor allows the analysis of a single CYP450 genotype per run [18,25]. Then, the DDI-Predictor algorithm computes the AUC ratio (R_AUC_) and its 95% tolerance interval, as follows [26]:

$$R_{\mathrm{AUC}}=\frac{\mathrm{Drug}\;\mathrm{AUC}\;\mathrm{with}\;\mathrm{the}\;\mathrm{studied}\;\mathrm{genotype}}{\mathrm{Drug}\;\mathrm{AUC}\;\mathrm{with}\;\mathrm{the}\;\mathrm{wild}-\mathrm{type}\;\mathrm{genotype}}$$

In the present study, the dose–adjustment ratio (R_d_, calculated by dividing the dose level prescribed before the PG test (corresponding to step 1 in Figure 1) by the clinically effective dose during the hospital stay (corresponding to step 4 in Figure 1) was calculated as follows:

$$R_{d}=\frac{\mathrm{Initial}\;\mathrm{dose}\;\mathrm{of}\;\mathrm{psychotopic}\;\mathrm{drug}}{\mathrm{Effective}\;\mathrm{level}\;\mathrm{after}\;\mathrm{adjustment}}$$

All patients for whom the drug regimen was maintained after the PG test were included. Patients were excluded in the following cases: (i) missing data on treatment optimization; (ii) the presence of other intrinsic or extrinsic factors that cannot be integrated into DDI-Predictor (even though they can be responsible for a variation in drug response); (iii) metabolic status not available in DDI-Predictor; and (iv) the presence of two or more genetic abnormalities (as mentioned above, DDI-Predictor allows the analysis of a single CYP450 genotype per run). In order to determine the value of DDI-Predictor, R_d_ was compared with R_AUC._

### 4.7. Data Presentation

Descriptive analyses were performed using GraphPad Prism^®^ software (version 7, GraphPad Software LLC, San Diego, CA, USA). Quantitative variables are quoted as the mean, range, and median. Qualitative variables are quoted as the frequency (percentage).

### 4.8. Ethics Approval

All data were anonymized and entered in an Excel^®^ 2013 spreadsheet (Microsoft Corporation, Redmond, WA, USA) spreadsheet. The study’s protocol was approved by a hospital committee with competency for research, and did not require approval by an institutional review board (Lille University Hospital, Lille, France; numbers: ID210 and 981). Written, informed consent from the patient was required for each PG test. The test order and the signed consent form were sent to the central laboratory.

## 5. Conclusions

Both TDM and PG testing can help the clinician to adjust psychotropic drug regimens. The systematic analysis of *CYP2D6* and *CYP2C19* genotypes may help to optimize patient management. A multidisciplinary interpretation of the TDM and PG results is probably essential for optimizing drug management for these complex patients, and to develop tools and guidelines on decision support for psychotropic drug prescription.

## Figures and Tables

**Table 1:** Plasma psychotic drug levels after TDM for the 27 patients with a PG test.

Indication for TDM	TDM Result			
	Total (*n* = 27)	Subtherapeutic Drug Level (*n* = 15)	Optimal Drug Level (*n* = 2)	Supratherapeutic Drug Level (*n* = 10)
Lack of a response at the therapeutic dose (suggested dose adjustment or drug switch)	18 (67%)	11 (61%)	1 (6%)	6 (33%)
Potentially poor adherence	5 (18%)	3 (75%)	0 (0%)	2 (25%)
Suboptimal drug tolerance	4 (15%)	1 (25%)	1 (25%)	2 (50%)

**Table 2:** Changes in psychotropic drug treatments prompted by PG testing. PM: poor metabolizer; IM: intermediate metabolizer; EM: extensive metabolizer; UM: ultrarapid metabolizer. When a new drug was not administered orally, the administration route is indicated as IM (intramuscular) or SC (subcutaneous).

Orally Administered Psychotropic Drugs Related to the PG Testing	Reason for PG Testing	CYP Isoform Studied and Phenotype	Expected Plasma Concentration, According to the Genotype Analyzed	Dose/Drug Adjustment after the Metabolic Status Result	Major CYP Isoform Responsible for Metabolism of New Treatment
The metabolic status could explain the treatment failure in 12 of the 30 cases (40%)					
Clozapine	High drug concentration (*n* = 1)	CYP2D6 IM, CYP1A2 IM	Elevated plasma concentration	* Dose reduction (from 200 mg to 175 mg)	
	Lack of a response at the therapeutic dose (*n* = 1)	CYP1A2 UM	Low plasma concentration	* Switch to haloperidol (SC)	3A4; 2D6
Clozapine and aripiprazole	Suboptimal tolerance; high concentration of aripiprazole (*n* = 1)	CYP2D6 IM, CYP4A5 IM	Elevated plasma concentration	No change	
Olanzapine	Low drug concentration (*n* = 1)	CYP1A2 UM, CYP2D6 IM	Low plasma concentration	Dose increase (from 40 mg to 60 mg)	
Olanzapine and aripiprazole	Suboptimal tolerance; high concentration (*n* = 1)	CYP2D6 IM, CYP4A5 IM	Elevated plasma concentration	Split the daily dose (from 20 mg 1/d to 10 mg × 2/d)	
Paroxetine	High drug concentration (*n* = 4)	CYP2D6 PM (*n* = 1)	Elevated plasma concentration	Switch to sertraline	2C19
		CYP2D6 IM (*n* = 3)		Dose reduction (from 60 mg to 40 mg)	
				* Switch to citalopram (1/4)	2C19
				* Switch to sertraline (1/4)	2C19
Quetiapine	Low drug concentration (*n* = 1)	CYP3A5 EM (partial explanation)	Low plasma concentration	No change	
Risperidone	Suboptimal tolerance (*n* = 1)	CYP2D6 IM	Elevated plasma concentration	Dose reduction (from 2 mg to 1.5 mg)	
Venlafaxine	Suboptimal tolerance (*n* = 1)	CYP2D6 IM	Elevated plasma concentration	Switch to escitalopram	2C19
The metabolic status could not explain the treatment failure and TDM results in 18 of the 30 cases (60%)					
Clozapine	Lack of a response at the therapeutic dose and high drug concentration (*n* = 1)	CYP1A2 UM (smoker)	Low plasma concentration	Tobacco stop suggested	
	Non-response at therapeutic doses (*n* = 1)	CYP1A2 UM (non smoker)	Low plasma concentration	No change	
Clozapine and escitalopram	Low drug concentration (*n* = 1)	CYP2D6 IM CYP3A5 IM	Elevated plasma concentration	Switch to clozapine and clomipramine	1A2; 3A4
Fluoxetine (+1: unknown)	Lack of a response at the therapeutic dose (*n* = 2)	None (*n* = 2)	No variation	Switch to amitriptyline	2C19; 2D6
				Switch to clomipramine	2C19
Paroxetine	High drug concentration (*n* = 1)	None	No variation	* Switch to venlafaxine	2D6
Quetiapine	Low drug concentration (*n* = 6)	CYP2D6 IM	Elevated plasma concentration	No change (*n* = 2)	
		CYP2D6 IM, CYP2C19 IM, CYP3A5 IM		* Switch to lithium carbonate	none
		None (*n* = 3)	No variation	* Switch to amisulpride	none
				Switch to olanzapine	1A2
				Switch to amisulpride	none
Risperidone	Lack of a response at the therapeutic dose (*n* = 2)	CYP1A2 UM (smoker) CYP2D6 IM	Elevated plasma concentration	* Switch to paliperidone (IM)	none
		None	No variation	* Switch to paliperidone (IM)	none
	Low drug concentration (*n* = 4)	CYP2D6 IM (*n* = 2)	Elevated plasma concentration	No change	
		CYP2D6 EM (*n* = 2)	No variation	Switch to olanzapine IM	1A2
				No change	

Result with * if the optimization drug was performed before the PG result.

**Table 3:** Comparison between the dose level after adjustment and the plasma drug concentration ratio predicted by DDI-Predictor. R_d_ corresponds to the ratio between the initial dose level and the effective dose level; R_AUC_ corresponds to the predicted ratio between the drug’s AUC with the patient’s genotype and the drug’s AUC with the wild-type genotype. All of the dose level adjustments (as expressed by R_d_) were consistent with the doses predicted by DDI-Predictor (i.e., the R_AUC_).

Psychotic Drug	Genotype *X/*X	R_d_	R_AUC_ with Tolerance Interval (5th to 95th Percentiles)
Paroxetine	CYP2D6*1/*4 or *2/*4	60/40 = 1.5	1.6 [1.09–2.36]
Quetiapine	CYP2D6*1/*2	1200/1200 = 1	1 [0.76–1.32]
	CYP2D6*1/*4 or *2/*4	900/900 = 1	1.09 [0.82–1.45]
Risperidone	CYP2D6*1/*2	4/4 = 1	1 [0.76–1.32]
	CYP2D6*1/*4 or *2/*4	2/1.5 = 1.33	1.66 [1.11–2.48]
Olanzapine and aripiprazole	CYP2D6*1/*4 or *2/*4	20/20 = 1 20/20 = 1	1.09 [0.82–1.45] 1.22 [0.9–1.66]

## Data Availability

Data is contained within the article and Supplementary Material.

## Supplementary Materials

The following supporting information can be downloaded at: https://www.mdpi.com/article/10.3390/ph17010021/s1, Supplemental Data: Isoforms of CYP450 involved in the metabolism of drugs used to treat psychiatric diseases avalaible on the French Market. Data obtained from Hiemke and al. 2017 and eVidal (september 2022). The highlighted isoforms are those routinely studied in the laboratory of our hospital.

## Abbreviations

AGNP	Arbeitsgemeinschaft für Neuropsychopharmakologie und Pharmakopsychiatrie
AP	antipsychotic
ATDs	antidepressants
AUC	area under the curve
CYP	cytochrome P450 superfamily
DDI	drug–drug interaction
EM	extensive metabolizer
IM	intermediate metabolizer
PG	pharmacogenetic
PM	poor metabolizer
TDM	therapeutic drug monitoring
UM	ultra-rapid metabolizer
