# Supplementary material for: Therapeutic Drug Monitoring and Pharmacogenetic Testing as Guides to Psychotropic Drug Dose Adjustment: An Observational Study
Source: Pharmaceuticals (Basel). 2023 Dec 22;17(1):21. doi: 10.3390/ph17010021 (PMC10818858; doi:10.3390/ph17010021)
Supplement: Supplementary file 1 [file pharmaceuticals-17-00021-s001.zip › pharmaceuticals-2671566-supplementary.pdf]

Supplemental data. Isoforms of CYP450 involved in the metabolism of drugs used to treat psychiatric diseases avalaible on the French Market. Data obtained from Hiemke and al. 2017 and eVidal (september 2022). The highlighted isoforms are those routinely studied in the laboratory of our hospital.

**Drugs**	**Isoforms of CYP450 involved in the metabolism**							
	**CYP1A2**	**CYP2A6**	**CYP2B6**	**CYP2C8**	**CYP2C9**	**CYP2C19**	**CYP2D6**	**CYP3A4**
Agomelatine	X					X		X
Alprazolam								CYP3A4 /5
Amisulpride	None							
Amitriptyline	X				X	X	X	X
Aripiprazole							X	X
Bromazepam						X		X
Buspirone								X
Carbamazepine	X			X				CYP3A4 /5
Chlordiazepoxide								X
Chlorpromazine	X						X	
Citalopram						X	X	X
Clobazam						X		X
Clomipramine	X					X	X	X
Clorazepat						X		X
Clozapine	X					X		X
Cyamemazine	X			X	X	X		X
Diazepam			X			X		X
Dosulepin						X	X	
Doxylamine	Unknown							
Duloxetine	X						X	
Escitalopram						X	X	X
Fluoxetine			X		X	X	X	
Flupenthixol							X	
Fluvoxamine	X						X	
Haloperidol							X	X
Imipramine	X					X	X	X
Lamotrigine	None							
Levomepromazine								CYP3
Lithium	None							
Lorazepam	None							
Loxapine	X			X		X	X	X
Maprotiline	X						X	
Melatonin	X							
Mianserine	X						X	X
Milnacipran								X
Mirtazapine	X						X	X
Moclobemide						X	X	
Nitrazepam								X
Olanzapine	X						X	
Oxazepam	None							
Paroxetine							X	X
Pimozide	X						X	X
Pipamperone	Unknown							

Prazepam						X		X
Promethazine							X	
Quetiapine							X	X
Risperidone							X	X
Sertraline			X		X	X	X	X
Sulpiride	None							
Tianeptine	None							
Tiapride	None							
Trimipramine					X	X	X	X
Valproic acid		X	X		X	X		
Venlafaxine					X	X	X	X
Vortioxetine		X			X		X	X
Zolpidem	X				X			X
Zopiclone				X				X
Zuclopenthixol							X	
